# Surgical treatment for brachiocephalic artery aneurysm with Takayasu arteritis using isolated cerebral perfusion: a case study

**DOI:** 10.1186/s13019-021-01413-1

**Published:** 2021-03-20

**Authors:** Kayo Sugiyama, Hirotaka Watanuki, Yasuhiro Futamura, Masaho Okada, Satoshi Makino, Katsuhiko Matsuyama

**Affiliations:** Department of Cardiac Surgery, Aichi Medical University Hospital, 1-1 Yazakokarimata, Nagakute, Aichi 480-1195 Japan

**Keywords:** Takayasu arteritis, Brachiocephalic artery aneurysm, Isolated cerebral perfusion

## Abstract

**Background:**

Takayasu arteritis (TA) is a chronic inflammatory disease that induces stenosis, occlusion, or aneurysmal degeneration of the aorta and its major branches. Though rarely reported, proximal aneurysmal lesions from the aortic root to the arch are more common in Asian populations than in Western populations. In the surgical treatment of TA, anastomotic aneurysm can be problematic. Furthermore, atherosclerotic complications should be considered in surgical treatment for elderly TA patients.

**Case presentation:**

Here, we report a case of brachiocephalic artery (BCA) aneurysm with TA for which surgical treatment was successful. Though it was solely a lesion of the brachiocephalic artery, after considering the patient’s clinical background and the features of TA, we chose a partial arch replacement. Further, for avoidance of anastomotic aneurysm, both distal and proximal anastomosis were reinforced with Teflon felt strips. Preoperative computed tomography detected severe atherosclerotic changes in the arch vessels. The patient underwent partial arch replacement using isolated cerebral perfusion (ICP) for brain protection and recovered without any neurological deficits.

**Conclusions:**

In avoidance with anastomotic aneurysm, reinforcement of the anastomosis was introduced. ICP was effective for brain protection in case with severe atherosclerotic changes.

## Background

The pathologic feature of chronic inflammation in Takayasu arteritis (TA) leads to stenosis, occlusion, or aneurysmal degeneration of the aorta and its major branches [1]. Although it has been found that the involvement of arch vessels is detected more frequently in Japan [2, 3], there have been few reports describing the surgical treatment for arch vessel aneurysm in TA. Moreover, treating arch vessel aneurysms remains controversial [4]. Though less-invasive methods favor endovascular repair, treatment is rarely feasible owing to anatomical limitations [4]. While graft interposition and patch plasty with partial clamp have the advantage of avoiding cardiopulmonary bypass, they can result in anastomotic complications [4, 5]. Because of chronic inflammation on the aortic wall [6, 7], partial or total aortic arch repair with cardiopulmonary bypass is desirable for treating arch vessel aneurysm in TA. To prevent anastomotic aneurysms, reinforcement of the suture lines has been recommended [1], and suppression of active or persisting inflammation with corticosteroid is reported [1, 3].

Moreover, atherosclerotic changes become problematic in elderly TA patients. In aortic surgery for patients with severe atherosclerotic changes, brain protection is a priority. While many reports have described brain protection techniques during aortic arch repair, isolated cerebral perfusion (ICP) may be promising in these cases [8]. In this report, the surgical treatment of brachiocephalic artery (BCA) aneurysm focused on brain protection is discussed for elderly patients with TA who are on steroid treatment. Furthermore, the method of preventing anastomotic aneurysms, including surgical strategy and perioperative management, is described.

## Case report

**Fig. 1:**
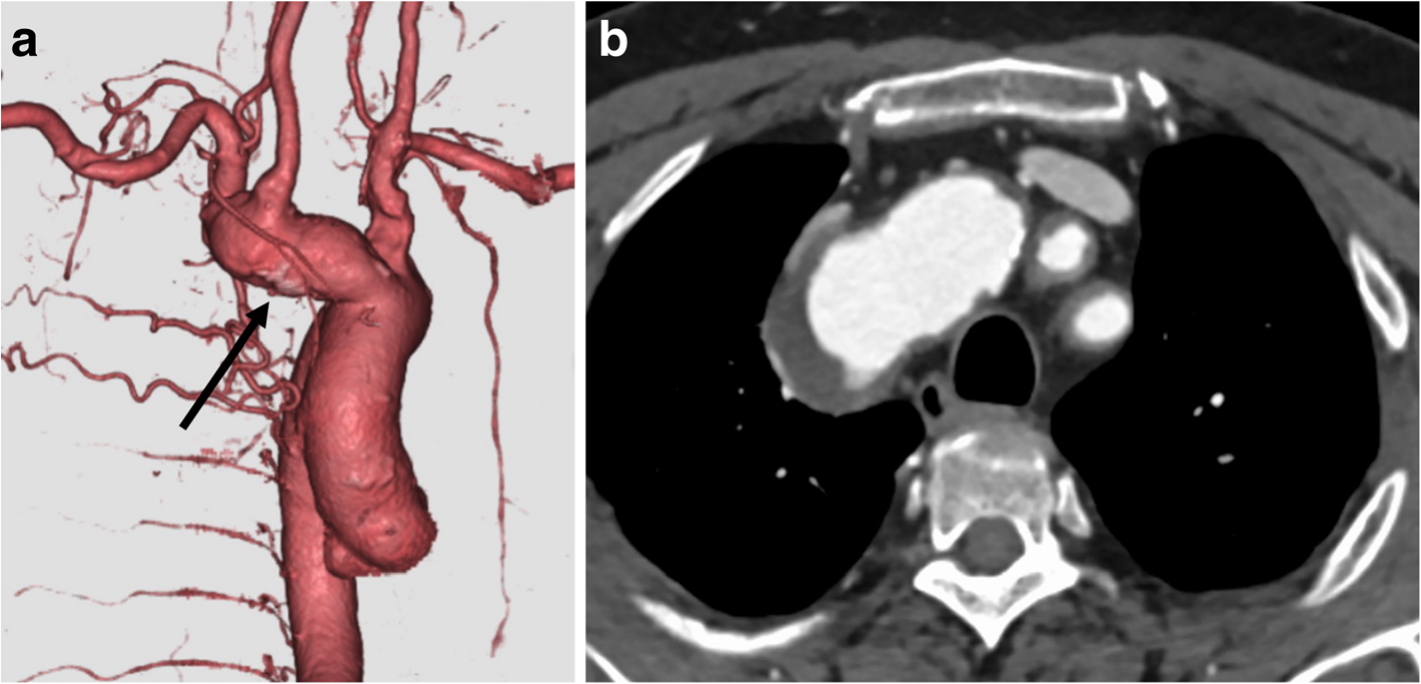
**a** Preoperative computed tomography showing brachiocephalic artery aneurysm (black arrow) measuring 32 mm in diameter with thickened aortic wall. **b** Preoperative computed tomography showing severe atherosclerotic changes in the aortic arch and orifice of the arch vessels

**Fig. 2:**
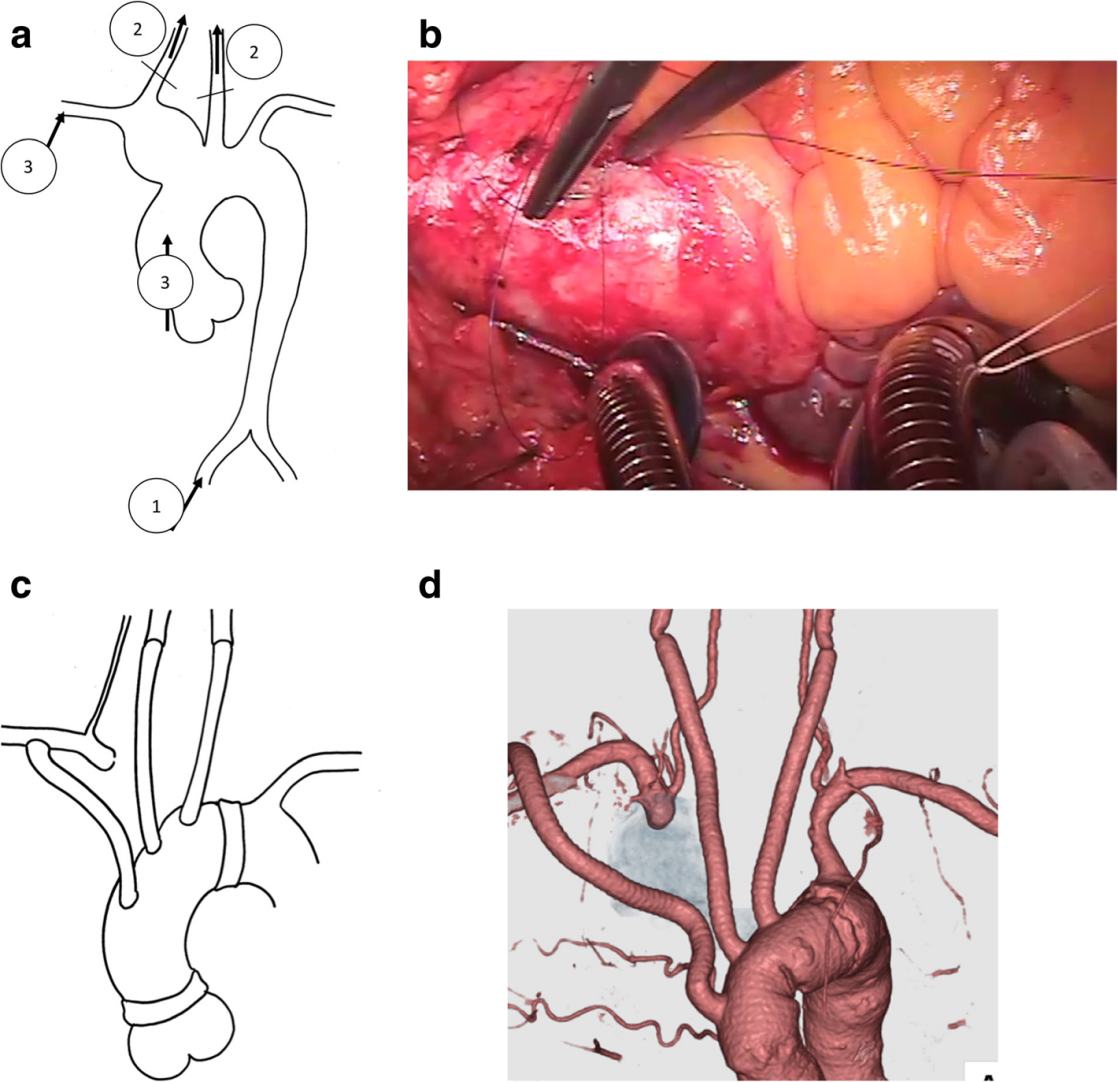
**a** Schematic drawing of the isolated cerebral perfusion technique. Black arrows indicate perfusion sites and the numbers representing the order of perfusion. **b** Intraoperative view showing thickening and hardness of the ascending aortic wall. **c** Completed drawing of partial arch replacement. **d** Postoperative computed tomography showing no anastomotic aneurysm and no abnormal flow in the previous aneurysm

A 78-year-old male patient with TA was referred to our hospital for the treatment of a BCA aneurysm. He had been treated for TA with 10 mg/day oral administration of prednisolone for 24 years. He was also treated for hypertension at a local hospital and had no history of cardiovascular diseases. He had moderate pulmonary emphysema due to a long history of smoking. Furthermore, though there were no obvious sequelae, he had a history of brain infarction. Computed tomography showed a BCA aneurysm measuring 32 mm in diameter with a thickened aortic wall ranging from the orifice of the BCA to the bifurcation of the right common carotid and right subclavian artery (Fig. 1a). Computed tomography also showed severely atherosclerotic changes in the arch vessels (Fig. 1b). C-reactive protein level was 0.2 mg/dl, and erythrocyte sedimentation rate was 6.0 mm/hour. Taking into consideration of the patient’s comorbidities as mentioned above, partial arch replacement with ICP was scheduled. Following median sternotomy, cardiopulmonary bypass was established with femoral artery cannulation (Fig. 2a) and bicaval drainage with a low flow (0.5 L/min). When the cardiopulmonary bypass got stabilized, direct cannulation of bilateral carotid arteries was added with a proximal clamp not to flush debris to the distal site (Fig. 2a). Total cardiopulmonary bypass was gained by additional cannulation through the ascending aorta and an 8-mm graft anastomosed to the right subclavian artery (Fig. 2a). The chronic inflammation changes compatible with TA, such as wall thickening and increased hardness, were observed in the ascending aorta (Fig. 2b). When the body temperature reached to 25 °C, lower body circulatory arrest was introduced. The orifice of the right subclavian artery was closed with a continuous 4–0 polypropylene suture through inside of the aneurysm. The orifice of arch vessels presented with severe atherosclerotic changes. Partial arch replacement with 28 mm quadrifurcated graft (Triplex®, Terumo Corporation, Tokyo, Japan) was performed with an open distal anastomosis technique. After distal anastomosis, the neck vessels including right subclavian artery, right carotid artery, and left carotid artery, were reconstructed with each branch of the artificial graft individually (Fig. 2c). An artificial graft anastomosed to the right subclavian artery was used for branch reconstruction (Fig. 2c, d). Both distal and proximal anastomosis were reinforced with Teflon felt strips at inner and outer side of the aortic wall. Weaning from cardiopulmonary bypass was uneventful, and the patient recovered without any neurological deficits. The time of operation, cardiopulmonary bypass, aortic cross-clamp, cerebral perfusion, and circulatory arrest were 442, 229, 128, 133, and 60 min, respectively. There were no significant changes in the continuous bilateral cerebral regional oxygen saturation values between the right and left side during the surgery as monitored using INVOS™ Cerebral/Somatic Oximetry Adult Sensors. Postoperative brain imaging detected no significant cerebral infarctions. Postoperative computed tomography demonstrated no anastomotic aneurysms and no abnormal flow in the aneurysm (Fig. 2d). Pathological results reported that the intima of the aorta had focal and raised plaques. During the perioperative period, management with additional prednisolone infusion was done, and the patient has never experienced episodes of exacerbation of inflammation. The patient remained free from any major adverse aortic and cerebrovascular events 1 year after surgery.

## Discussion

The main pathologic feature of TA is the extensive destruction of the medial elastic fibers that maintain the strength of the aortic wall [6, 7]. This feature often leads to stenosis, occlusion, or aneurysmal degeneration of the aorta and its major branches [1]. Asian populations tend to develop proximal or aneurysmal lesions from the aortic root to the arch at a higher frequency than Western populations [2, 3]. However, there have been few articles about surgical repair for arch vessel aneurysms in TA.

Operative indications for BCA aneurysms include either ruptured or symptomatic aneurysms, such as distal embolization or compression of the adjacent structures. When the aneurysms are saccular or when their maximum transverse diameter is more than 3 cm, surgical repair should be considered, even when asymptomatic [4]. Although the present case did not present with any symptoms related to the aneurysm, a size of over 3 cm was the deciding factor for surgical treatment.

Treatment strategy for BCA aneurysm depends on the extent of involvement. When the lesion is extremely localized, endovascular repair can be performed, but this method is rarely enforceable [4]. Mostly open surgical repair including graft interpose, patch plasty with partial clamp, and partial or total aortic arch repair has been reported [4]. Patch plasty with partial clamp seemed desirable because circulatory arrest would have been avoided; however, anastomotic problems remained. Okita et al. reported a higher incidence of pseudoaneurysm or residual aneurysmal formation after patch repair for a saccular aneurysm of the aortic arch [5].

Furthermore, in TA, it often happens that the aorta has been affected by chronic inflammation, even though the preoperative images appeared to show a healthy and normal aorta. Because a serious long-term complication in TA includes anastomotic aneurysms, sites of normal tissue without inflammatory changes should be chosen as the anastomotic sites [6]. It is also important to exclude inflamed cervical vascular lesions [3]. In accordance with these reports, reinforcement of the sutures has been recommended [1]. Even for routine aortic surgery, reinforcement of the sutures with felt strips has been recommended [9, 10]. Though felt strips can be a source of infection or anastomotic stenosis, it is indispensable as reinforcement, especially for a vulnerable aortic wall resulting from acute aortic dissection, connective tissue disease, and TA [9, 10]. In the present case, reinforcement of the proximal and distal anastomosis with Teflon felt strips at inner and outer side of aortic wall was performed. Because an anastomotic aneurysm may occur at any time after surgery, regular follow-up using multiple imaging modalities is mandatory [7]. Although systemic inflammation or steroid administration had reportedly little influence on formation of anastomotic aneurysm [6, 7], suppression of active or persisting inflammation with corticosteroids is recommended [1, 3].

The indications for endovascular repair of an aortic arch aneurysm have been expanded, and the outcomes continue to improve. Successful endovascular aneurysmal repair for dilated lesions due to aortitis has been reported [11]. However, reintervention for a ruptured stent-graft and new aneurysm formation after endovascular treatment was also described [12]. In the present case, open surgical repair was chosen because there were no anatomical indications for thoracic endovascular aortic repair, and inflammatory changes in the aortic wall were a concern. Since few reports describe the stable outcome of stent-graft in TA, its indication should be carefully considered.

In aortic surgery for patients with severe atherosclerotic changes, protection of the brain tissue is important. There have been many reports describing various strategies for brain protection; however, the optimal management remains unclear. Axillary or BCA perfusion seems promising, but turbulent flow in the proximal aortic arch may still dislodge the atheroma [8]. Furthermore, bilateral axillary artery perfusion may fail to reduce the risk of embolism of the left common carotid artery because blood flow from the axillary arteries spreads into the aortic arch, resulting in plaque disruption by jet flow [13]. Shiiya et al. introduced an isolation technique to address these issues [8]. This technique involves the concept of distributing blood flow to each neck vessels individually, resulting in the prevention of a distal embolism. Kasama et al. reported the advantage of this method by investigating ICP in elderly patients with severely atherosclerotic changes [13]. In the present case, this strategy enabled us to avoid jet flow from the aortic arch, and the patient recovered without any neurological deficits. Slowly establishment of the cardiopulmonary bypass through a femoral artery and additional carotid artery perfusion could prevent plaque embolism from the aortic arch. Further studies on the effectiveness of ICP are warranted.

## Conclusions

We present the case of BCA aneurysm with TA that was successfully treated with partial arch replacement. In avoidance with anastomotic aneurysm, reinforcement of the anastomosis was introduced. ICP was effective for brain protection in case with severe atherosclerotic changes.

## Data Availability

All data generated or analyzed during this study are included in this article.

## Abbreviations

TA: Takayasu arteritis
BCA: Brachiocephalic artery
ICP: Isolated cerebral perfusion
